# Neutrophil-to-Lymphocyte Ratio Predicts Prognosis in Castration-Resistant Prostate Cancer Patients Who Received Cabazitaxel Chemotherapy

**DOI:** 10.1155/2017/7538647

**Published:** 2017-08-29

**Authors:** Koichi Uemura, Takashi Kawahara, Daisuke Yamashita, Ryosuke Jikuya, Koichi Abe, Tomoyuki Tatenuma, Yumiko Yokomizo, Koji Izumi, Jun-ichi Teranishi, Kazuhide Makiyama, Yasushi Yumura, Takeshi Kishida, Koichi Udagawa, Kazuki Kobayashi, Yasuhide Miyoshi, Masahiro Yao, Hiroji Uemura

**Affiliations:** ^1^Departments of Urology and Renal Transplantation, Yokohama City University Medical Center, Yokohama, Japan; ^2^Department of Urology, Yokohama City University Graduate School of Medicine, Yokohama, Japan; ^3^Department of Urology, Yokosuka Kyosai Hospital, Yokosuka, Japan; ^4^Department of Urology, Kanagawa Cancer Center, Yokohama, Japan; ^5^Department of Urology, Hiratsuka Kyosai Hospital, Hiratsuka, Japan

## Abstract

**Introduction and Objectives:**

An elevated neutrophil-to-lymphocyte ratio (NLR) has been suggested to be associated with a poor prognosis in several cancers. We evaluated the utility of an elevated NLR as a biomarker to predict the prognosis of metastatic castration-resistant prostate cancer (mCRPC) patients treated with cabazitaxel (CBZ).

**Methods:**

We analyzed 47 patients who received CBZ chemotherapy for mCRPC in our institutions. The NLR was calculated using the neutrophil and lymphocyte counts before CBZ chemotherapy. We determined the NLR cut-off value based on the sensitivity and specificity levels derived from area under the receiver operator characteristic curves for death. A multivariate analysis was performed to investigate the association between the NLR and the prognosis.

**Results:**

The median overall survival (OS) after CBZ was 10.0 months (range: 6.3–13.2). The median OS was shorter in patients with a high NLR (≥3.83) than in those with a low NLR (<3.83) (5.8 versus 13.2 months, *p* = 0.018). In the multivariate analysis, the NLR, patient age, and lymph node (LN) metastasis were independent predictors of the OS (hazard ratio 3.01, *p* = 0.030; hazard ratio 3.10, *p* = 0.029; hazard ratio 12.38, *p* = 0.001, resp.).

**Conclusions:**

NLR might be a useful prognostic biomarker in mCRPC patients treated with CBZ.

## 1. Introduction

Prostate cancer is the most common cancer and its incidence has markedly increased in recent years [1]. Androgen deprivation therapy (ADT) is the standard treatment for advanced or metastatic prostate cancer. Despite the higher response rate of ADT, most patients develop castration-resistant prostate cancer (CRPC) [2, 3]. Several treatments, including enzalutamide (ENZ) [4, 5], abiraterone acetate (AA) [6, 7], docetaxel (DOC) [8, 9], cabazitaxel (CBZ) [10], radium-223 [11], and sipuleucel-T [11], have been shown to prolong overall survival (OS) in patients with metastatic CRPC (mCRPC).

CBZ is the first chemotherapeutic agent to prolong OS in mCRPC patients after docetaxel. The TROPIC study showed that CBZ prolonged OS in comparison to mitoxantrone (15.1 months versus 12.7 months) and that it reduced the relative risk of death by 30% (HR: 0.70, *p* < 0.001). Based on that study, CBZ has been widely accepted as a new-generation cytotoxic systemic chemotherapy for mCRPC. Although some prognostic factors and biomarkers have been reported, no clinically available biomarkers have been established for CBZ treatment.

Inflammation plays an important role in the development and progression of cancer. The neutrophil-to-lymphocyte ratio (NLR) has been suggested as a simple marker of the systemic inflammatory response and can be easily measured from routine complete blood counts (CBCs) in the peripheral blood. The NLR has been reported to be an independent prognostic factor in several cancers, including prostate cancer. This study examined the correlation between NLR and the prognosis of CRPC patients who were treated with CBZ.

## 2. Materials and Methods

### 2.1. Study Design, Patients, and Treatments

We retrospectively analyzed a total of 47 patients who received CBZ for the treatment of mCRPC in our institutions from 2014 to 2016. All of the patients had pathologically confirmed prostate carcinoma. Among these 47 patients, 37 had metastatic prostate cancer at the time of the diagnosis. All of the patients were initially treated with ADT, which was changed to antiandrogen therapy after treatment failure, followed by docetaxel with dexamethasone or ENZ/AA plus prednisolone.

CBZ was intravenously administered at a dose of 20 mg/m^2^ over 1 h on day 1 in triweekly cycles with continuous oral prednisone (10 mg/day). Dose modification was allowed according to the patient's condition, patient age, or bone marrow suppression. The treating physician could decide to stop the therapy at any time based on the patient's condition, PSA progression, or objective evidence of tumor progression, such as computed tomography or bone scintigraphy.

### 2.2. The Clinical and Laboratory Assessments

CBCs were performed and NLR was calculated using the neutrophil and lymphocyte counts obtained on the same day or a few days before the initiation of CBZ chemotherapy. No patients had any bacterial or viral infections at the induction of CBZ. We determined the cut-off point of the NLR based on the sensitivity and specificity levels derived from the area under receiver operator characteristic (AUROC) curves, which were plotted using either disease progression or overall mortality.

### 2.3. Statistical Analysis

The Mann–Whitney *U* test was used to analyze the patient characteristics and pretreatment factors. The Kaplan–Meier curve was used to estimate the distribution of survival. The log-rank test was used to analyze the differences in survival. The Cox proportional hazards model, with a stepwise regression analysis, was used to investigate the association between clinical variables at the time of the induction of CBZ. The variables included the PSA level, patient age, albumin (Alb), the lactate dehydrogenase (LDH) and alkaline phosphatase (ALP) levels, the courses of DOC, visceral or lymph node (LN) metastasis, the NLR, and OS after the induction of CBZ. LN metastasis was defined by a size of >15 mm based on the RECIST guidelines (version 1.1). The cut-off points for continuous variables (excluding the NLR) were determined based on the median value of each variable. All of the statistical analyses were performed using the GraphPad Prism software program (GraphPad Software, La Jolla, CA, USA). All tests were two-sided, and *p* values of* <0.05* were considered to indicate statistical significance.

## 3. Results

The patients' clinical characteristics are shown in Table 1. Based on the AUROC curves, the NLR cut-off value for the OS was determined to be 3.83. There were 20 and 27 patients in the NLR ≥ 3.83 and NLR < 3.83 groups, respectively. Cancer death occurred in 27 (57.4%) patients. The median OS after the induction of CBZ was 10.0 months (95% confidence interval (CI): 6.3–13.2 months).

**Figure 1:**
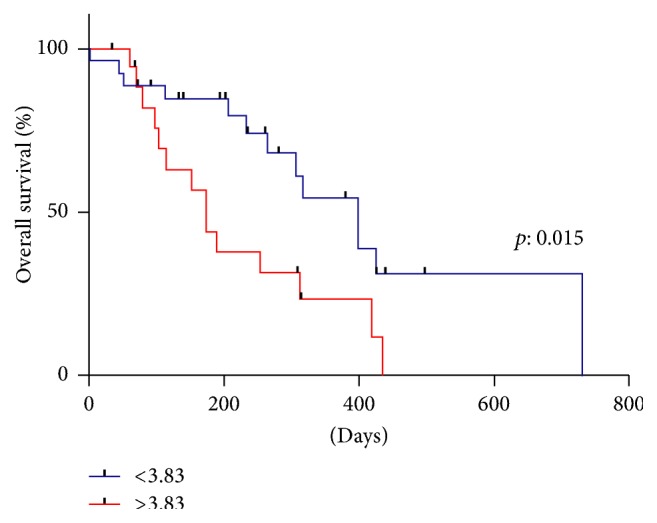
Kaplan–Meier curve for OS after CBZ according to the NLR. The median OS in patients with the NLR < 3.83 and NLR ≥ 3.83 were 13.2 months and 5.8 months.

We compared the survival probability according to the pretreatment NLR. The median OS in patients with an NLR of <3.83 was 13.2 months, while that in patients with an NLR of ≥3.83 was 5.8 months. The Kaplan–Meier curves showed that a higher NLR was significantly correlated with the risk of mortality (*p* = 0.015; Figure 1).

The multivariate analysis of 9 factors identified the NLR, patient age, and LN metastasis as independent prognostic biomarkers for OS after the induction of NLR (≥3.83 versus <3.83; HR: 3.01; 95% CI: 1.06–8.49; *p* = 0.030), patient age (≥71.4 versus <71.4; HR: 3.10; 95% CI: 1.11–8.63; *p* = 0.029), and LN metastasis (yes versus no; HR: 12.38; 95% CI: 2.62–58.35; *p* = 0.001) in Table 2.

**Figure 2:**
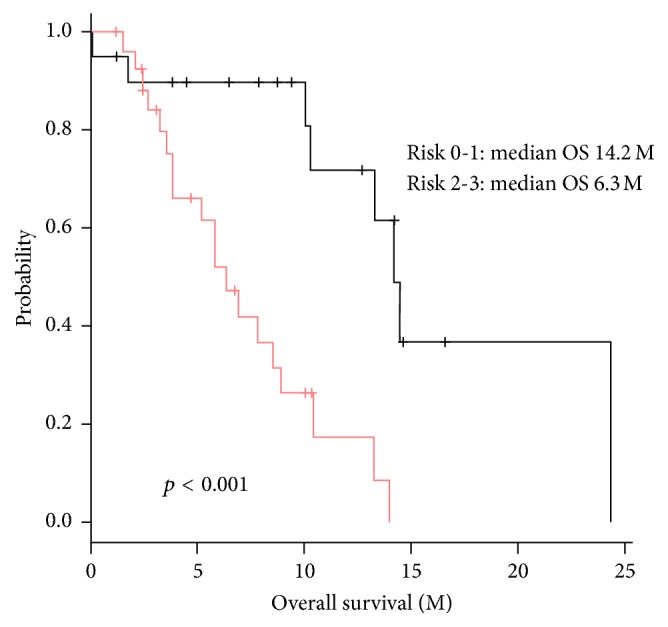
Kaplan–Meier curve for OS after CBA according to the risk group. We stratified the patients into two cohorts with low risk (0-1 risk factor) and high risk (2-3 risk factors). The median OS of patients in low and high risk were 14.2 months and 6.3 months.

The patients were classified into a low-risk group (0-1 risk factor) and a high-risk group (2-3 risk factors) based on the number of risk factors that they possessed. The characteristics of the two groups are shown in Table 3. Kaplan–Meier curves showed that the survival of the high-risk group after the induction of CBZ was significantly poorer than that of the low-risk group (median OS: 14.2 months versus 6.3 months; *p* < 0.001) (Figure 2).

## 4. Discussion

In the present study, we reported the usefulness of the NLR as a prognostic factor in mCRPC patients who were treated with CBZ. Since the TROPIC study, CBZ has widely been used as a new-generation cytotoxic systemic chemotherapy for cases in which docetaxel is ineffective. The landscape for the treatment of mCRPC might be gradually changing with the development of hormonal therapies (AA or ENZ), radiopharmaceutical agents (radium-223), cytotoxic chemotherapies (DOC or CBZ), and immunotherapy (sipuleucel-T). Several phase III trials showed that these agents could improve the survival of CRPC patients, while many studies have reported these agents to be useless. CBZ might be regarded as the last agent to prolong the OS of mCRPC patients who show progression after DOC in Japan. However, hematological adverse events, such as neutropenia, febrile neutropenia, and anemia, frequently appear during CBZ therapy. Thus, useful and reliable biomarkers that provide additional prognostic information in relation to CBZ treatment are needed to assist physicians in making decisions regarding the timing of induction or discontinuation and dosage adjustment. The reasons of CBZ dose reduction from 25 mg/m^2^ to 20 mg/m^2^ were dependent on the high incidence of febrile neutropenia in Japanese patients [12].

It has been proposed that the NLR can be used to estimate the magnitude of systemic inflammation in cancer patients [13–16]. Several retrospective studies have evaluated the baseline NLR as a prognostic factor in prostate cancer [17–20]. The NLR can easily be calculated from the CBC data without much labor or cost. Thus, the NLR is a useful tool when considering changes in the treatment of CRPC patients undergoing CBZ chemotherapy.

Various mechanisms have been suggested to lead to epithelial-to-mesenchymal transition, including an increased supply of growth factors, survival factors, proangiogenic factors, extracellular matrix-modifying enzymes (which can facilitate invasion and metastasis), and inductive signals [21, 22]. Cho et al. reported that patients with an elevated NLR exhibit a relative lymphocyte-mediated immune response to malignancy, thereby worsening their prognosis and increasing the potential for tumor progression [23, 24]. However, none of the mechanisms reported to underlie the link between the NLR and cancer progression have been widely accepted.

Our study is associated with some limitations. First, this study was retrospective in nature. As a result, the data for some of the variables were missing. Second, our population was relatively small and the observation period was relatively short. The evaluation of a larger patient population over a longer time is needed to verify the NLR as a prognostic factor. Finally, our definition of lymph node metastasis was not clear. We divided the patients into two groups. Multiple lymph node metastasis was not equal to simple metastasis; however, it was difficult to describe the volume of the metastatic lymph nodes as an objective and quantitative measure.

Despite the effectiveness of new agents including AA, ENZ, Ra-223, and CBZ, most patients developed resistance. Some new agents such as olaparib, pembrolizumab, and ipilimumab will be come to clinical use. In that situation, NLR might be a new factor to select these drugs [25–27].

In conclusion, we demonstrated that the NLR might be a new biomarker that can be used to predict the prognosis of mCRPC patients who are treated with cabazitaxel.

## Figures and Tables

**Table 1:** Patient characteristics (*n* = 47).

Variables	Total	NLR < 3.83	NLR ≥ 3.83	*p* value
Number	47	27	20	
Age (years)	71.4 (71.0 ± 7.0)	71.1 (71.0 ± 6.2)	72.1 (71.0 ± 8.2)	0.598
Baseline PSA (ng/mL)	150.3 (461.2 ± 789.9)	164.6 (478.4 ± 754.6)	126.9 (438.1 ± 854.7)	0.475
Parts of metastasis				
Bone	47 (100.0%)	27 (100.0%)	20 (100.0%)	
Lymph nodes	31 (65.9%)	17 (62.9%)	14 (70.0%)	0.624
Visceral	22 (46.8%)	10 (37.0%)	12 (60.0%)	0.124
Alb (mg/dL)	3.7 (3.6 ± 0.5)	3.8 (3.7 ± 0.4)	3.4 (3.4 ± 0.6)	0.110
LDH (IU/L)	269 (398.7 ± 496.7)	231 (399.7 ± 641.1)	362 (397.3 ± 186.5)	0.026
ALP (IU/L)	429 (794.9 ± 902.0)	373 (745.6 ± 855.8)	453 (861.6 ± 979.5)	0.297
Courses of docetaxel	9 (12.6 ± 11.1)	9 (11.3 ± 10.8)	7 (13.1 ± 10.9)	0.517

**Table 2:** Multivariate analysis of overall survival after the administration of cabazitaxel.

Multivariate analysis	HR	95% CI lower	95% CI upper	*p* value
Visceral metastasis	0.52	0.16	1.61	0.260
PSA ≥ 150.3 or <150.3	1.45	0.49	4.20	0.497
NLR ≥ 3.83 or <3.83	3.01	1.06	8.49	0.030
Alb < 3.7 or ≥3.7	2.45	0.81	7.34	0.108
Age ≥ 71.4 or <71.4	3.10	1.11	8.63	0.029
DOC course ≥ 9 or <9	2.12	0.71	6.33	0.177
Lymph node metastasis	12.38	2.62	58.35	0.001
LDH ≥ 269 or <269	2.85	1.03	9.31	0.052
ALP ≥ 429 or <429	0.80	0.26	2.36	0.108

**Table 3:** Distribution of risk classified by risk group.

	Low risk	High risk
Risk factors, *n*	0-1	2-3
Patients, *n* (%)	20 (42.5%)	27 (57.5%)
NLR ≥ 3.83, *n* (%)	2 (10.0%)	18 (66.7%)
Age ≥ 71.4 years, *n* (%)	4 (20.0%)	20 (74.0%)
Lymph node metastasis, *n* (%)	8 (40.0%)	23 (85.2%)

## Additional Points

*Availability of Data and Materials.* Due to ethical restrictions, the raw data that was used in this study is available upon request from the corresponding author.
